# Fourth Annual Meeting of the State Society of Arkansas

**Published:** 1879-06

**Authors:** 


FOURTH ANNUAL MEETING OF THE STATE MEDICAL SOCIETY OF ARKANSAS, was held at Little Rock, May 22d, 1879, and was largely attended. Addresses of welcome was given by GEORGE C. HART, M. D., and was responded to by Dr. DALE, after which came the Annual Address of the President, Dr. A. A. HOMER, of Helena.

From report in the Arkansas *Democrat* of Little Rock, we infer that the Profession in Arkansas are now in prosperous condition, showing great activity and strength.

"In the matter of the appointment of a committee by the Society to act in the capacity of a State Board of Health, and to co-operate with the Mississippi Valley Inter-State Sanitary Commission, your committee is of the opinion that the recommendation is a good one, and would offer, in furtherance of this idea, the following:

*Resolved*, That the President of the Society appoint nine members of the same, who shall act in the capacity of a State Board of Health as far as practicable, and under the sanction and co-operation of the State government.

That said committee or State Board of Health shall organize by the election of a President and Secretary, and shall endeavor to secure membership in the Mississippi Valley Inter-State Sanitary Commission on the same footing as the Sta e Boards of Health."

